# Editorial Department

**Published:** 1882-04

**Authors:** 


### The Bistoury.

ELMIRA, N. Y., APRIL, 1882.

The BISTOURY is published Quarterly, upon the first of April, July, October and January, at Fifty Cents a year in advance.

### Editorial Department.

THE ATHLETES.—We wonder whether rowing is not a Trickett which Hanlan is likely to fail, after all ?

PARK CHURCH DEBT.—The entire sum necessary to liquidate the remaining debt upon Park Church has been subscribed by members of that congregation. This magnificent edifice is now free from debt.

FROM CAMP.—We hope to greet our readers with a manifesto from camp, in the next number. We expect to spend our vacation in the usual manner, and at the same old spot that has afforded us recreation and amusement for the past eighteen years.

THE GAZETTE.—It is scarcely necessary to mention the fact, for all cannot have failed to observe that the number of the Daily Gazette, published on Saturday's, is made peculiarly attractive and entertaining by the contributions of the "Old Major" and the "Saturday Miscellany." Major H. H. Rockwell—"The Old Major" is rapidly becoming one of the most ready and attractive writers know to newspaper fame. We predict for him a brilliant future in a path manifestly well chosen.

CROWDED OUT.—By reason of the unusually full and interesting department devoted to contributions. Our Correspondence, in which is a very entertaining letter from Dr. Gleason, descriptive of alligator shooting in Florida, is crowded out. Also an article from Theophrastus Schopenhauer—a continuation of the school question, commenced in the October number. We are sorry that a lack of space prevented us from presenting all of the excellent articles furnished for this number. They will "keep" however, for the next one.

THE OPERA HOUSE in this city has outgrown its usefulness. It is neither convenient nor safe, while its filthiness is past endurance. When an actress sweeps the stage with the train of her dress, it is a signal for the audience to sneeze. This sort of applause is indigenous to the Elmira Opera House, and is caused by the accumulated dust that finds lodgement there from year to year. If the manager would only have the stage scrubbed, one might put up with the filth under foot, in the body of the house, a little while longer.

A new place for amusements is urgently required for our city—on the ground floor, and with all the modern improvements.

MICROSCOPICAL SOIREE.—The microscopical society of this city mean to give a reception to their friends, and to exhibit to them some of the beauties of that minute animal and vegetable life that has been so fascinating to them as to excite the wonderment of those not familiar with the use of the microscope. The entertainment will be given in the lecture room of Park Church on the evening of April 20th, from 7½ to 10 o'clock. Eminent microscopists from Philadelphia, New York, Rochester and other cities will join their Elmira brethren in the exhibition. Six hundred cards of admission only, will be issued. The Elmira Society pay the entire expense of the entertainment and receive their friends free of charge.

A NEW MOTTO.—We saw suspended in the dining room of a prominent member of the press, in this city, an illuminated motto expressing a bran-fired-new sentiment. We glanced at it several times without being able to decipher any of the usual wordings so displayed. We could not make it read—"God Bless Our Home"—"Give Us Our Daily Bread," nor "Home Sweet Home." Adjusting our spectacles therefore, we approached it and read :

"Don't Forget to Take Your Medicine !"

The host kindly explained that it was placed there more for real use than as a matter of ornamentation, since both he and his wife had been under a physician's care for some time and each had been in the habit of reminding the other to take the usual dose of medicine before every meal. This "motto" was therefore displayed upon the dining room wall as a gentle reminder to both.

OSCAR WILDE.—Æstheticism is all well enough when engaged in modestly, or at least unostentatiously. But when a semi-lunatic of the Oscar Wilde pattern, overwhelms us with all the æsthetic paraphernalia—sun-flowers, lillies and knee breeches—all at one fell swoop, it is more than even a "progressive chap," can endure. You see, there is such a thing as running this æsthetic nonsense "into the ground." We admire those people of really æsthetic tastes, but when it is exhibited by pinning blue ribbon bows on red apples to relieve monochromotism for instance, why, then, it becomes exasperating.

The advent of Oscar Wilde only substantiates the fact hitherto often demonstrated, that no matter how absurd a doctrine of religion, morals or fashion may be advocated by any intelligent "crank," he will gather to himself many sympathizers, and eventually a host of followers.

---

WELL FILLED.—Our contributors, we are glad to note, have occupied our pages so completely as to leave but little space for the editor. This circumstance not only makes our own labors light, but also affords our patrons most excellent reading. The usual writers for the BISTOURY—Mr. Pratt and Mr. Towner, whose articles we have reason to know are eagerly sought for, are supplemented in this number with a symposium upon a theme full of interest at this time and conducted by those well known and always entertaining writers, Drs. Knox and Beecher, of this city, and Mr. Jas. B. Chandler of Philadelphia. The first two gentlemen handle the subject with that vigor and originality for which they are both so celebrated, while Mr. Chandler brings to the subject an experience gleaned from a life-long mingling with those philanthropists who have made the inspection and improvement of prisons their life work. Three more competent gentlemen could not well be found to discuss the questions propounded in our symposium. We are sure that a careful reading of their articles will afford much opportunity for thought and reflection.

---

THE REFORMATORY INVESTIGATION.—Whatever the causes that led to this investigation, and however wise and proper the proceeding, the method of it is open if not to condemnation, at least to criticism. We see it stated that, while the investigating committee were passing through the prison in this city, recently, the prisoners were permitted to hand letters of complaint to the visitors, and to engage them freely in conversation, in which the prison officials were soundly abused. It would seem that such an uncalled for license to criminals must be indulged in greatly to the prejudice of good order and dicipline in the institution. Altogether too much sympathy and condolence is gushed upon the criminal classes. By far too many people are endeavoring to administer a punishment that does not punish—a a lashing that does not smart—an imprisonment that shall be fragrant with delicate missives and boquets. This meddlesome interference upon the part of perhaps well meaning but very silly people is doing much to make our methods of well deserved punishment inoperative. We have already seen evidences of its evil influences in our own city, where at least one brutal criminal sought to slay another that he "might receive boquets and be petted and admired by the ladies" as was another murderer who had just been tried. If sane people have not respect enough for themselves to refrain from such questionable practices, they should certainly be compelled to do so by the officers of the law, to the end that justice and the law might be satisfied.

---

DR. JAS. A. HALL.—Just why one should write a eulogy of the dead cannot be satisfactorily explained in few words. It rarely occurs that other than words of praise are expressed of those who have gone before. But, of Dr. Hall, in his life-time, everyone with whom he came in contact spoke in the most endearing terms, and, having upon more than one occasion recommended him to others for those sterling qualities most respected of men, it will not seem flattery to testify to a remembrance of his exalted manhood, now that he is dead.

For three years, Dr. Hall was in our employ as resident physician in the Surgical Institute, —an office most difficult and perplexing to fill. During that time, he won our lasting regard, admiration and love. His devotion to, and untiring zeal for the advancement of the interest of his employer, without consideration for himself, was a peculiar trait in his character. To his work, he brought a bright, cheerful disposition, was always merry, prompt, and had a hearty, joyful greeting for every one, while he was a gleam of sun-shine to the sick and suffering. In the person of Dr. Hall was embodied —we verily believe—*all* the qualities of manhood worth the having. Intelligent, honest, obliging, of engaging and winning manner and charming in conversation; above all, he was unwavering in his christian faith, and practiced the attributes of the christian gentleman with an unobtrusiveness that must have been as satisfactory and comforting to himself as it was most charming to his acquaintances. In his painful illness, which he recognized from the beginning as a speedily fatal one, he manifested more anxiety for the comfort of his cherished family,—carefully striving to conceal his own suffering that they might not be pained—than he did for his final end. He had a most sublime faith that permitted him to talk cheerfully to his family, advising them how to proceed after his death, and taught them by his wonderful example, that death was not such a dreadful affair after all. And so he passed away, cheerful, happy, contented—one of the bravest, gentlest souls that God ever blessed with immortality.

---

FRITZ VISITS PHILADELPHIA.—Fritz received an invitation from his big brother to visit him in Phila. He was not slow in first accepting the invitation and then laying plans to get consent and the necessary money from the "Govenor." He formed a powerful alliance with his mother to whom he made promises of future good behavior the fullfillment of which would put an arch angel to blush. Fritz laid his plans and carried them out with such consummate skill that he not only obtained permission to go but the necessary sum of money, as well, to carry out the expedition, before the old gentleman was fairly aware of it. So he started in high feather, reached his destination safely, and penned the following letter to his brother at home, in less than one week.

PHILA., March 18, 1882.

DEAR BROTHER.—I had an awful good supper at White Haven—they had cakes with raisins in 'em, so'd Mrs. D., at her house—and ice cream too, and it didn't make me sick neither. I've been up to where they cut folks up. [His brother, a medical student, took him to the dissecting room,] but they were all *dead* folks—they looked just like a whole lot of funerals—that's all. But I saw a live feller cut next day and he hollered awful. I've seen the electric lights up on poles that shine just like moons. I went to Wannamaker's big store—goodness! It's like a great big circus! I spent all my money there and had to borrow some more. Then, we went up the Delaware on a steam boat, and I went all over a ocean steamer and saw where they *eat*. I went across the river on a ferry boat that carried lots of big wagons and horses on it. I rode up on an elevator and couldn't find out what made it go. They wouldn't let me look out to see. I bought a toy pistol and lost it—I'm glad of it, I might have got the lockjaw, too. I've got two tops and a lot of marbles, and a magic lantern, and a man's bladder left. I'll show 'em to you and we'll blow it up when I get home. Howard thinks the bladder won't keep—but it will, for I put some diabolic acid on it, same as I saw pa put on his'n, at home. O, then, we went out to the Zoo and saw everything. I liked the monkeys best—don't you? We rode on the elevated rode—it peared awful funny riding right over the houses. All the boys rides bicycles down here. They go just as smooth. A feller told me his'n cost a hundred dollars. Let's save our pennies and get one too. There's lots of stores here—I walked as much as ten miles and nothing but stores all the way. I wonder where all these store-keepers live? I wouldn't want to keep store here, would you? I'd like to be a street-car conductor and shoot that funny pistol that rings a bell all the while. I asked Howard what he was shootin' at, and he just laughed. I've got ten cents left and I'll be home next week. I'll buy lots more things though—if I can get more money. Send me some of yourn. It's bed time, an' I'm awful tired, but I'm going to stay up to see the procession.

Yours, FRITZ.

---

THE GENESIS OF SKEPTICISM.—It seems to be pretty generally agreed upon that this is an age of skepticism, of doubt, of distrust, of *un*belief. The faith which could move mountains—yes, or even mole hills, is as much of a thing of the past as the Dodo. The orthodox bemoan and the heterodox applaud this condition of affairs, but both admit its existence. Our abnormally pious friends accuse indulgence in the fascinations of science, to the exclusion of the affairs of Church, as being the prime factor in bringing about this state of affairs. Let us, without siding with either party, simply accept it as an admitted fact, and inquire into the reasons which have produced such a remarkable change in the condition of minds of civilized humanity. Why are we prone to disbelief? Why is there no faith in us? Simply because our faith is educated out of us from our cradles up.

A hundred years ago, the story of Jack the Giant Killer or of Cindirella was told to wondering children by adults whose faith in the supernatural was scarcely less than that of their baby auditors. The boy or girl grew up, with faith unshaken by contact with crude. hard fact, to accept unquestioningly the wonders of fairy land, the mystery of the basketed baby from Heaven, etc.

Witchcraft was an ever-present reality. Grave judges and right reverend prelates discussed learnedly upon the power of the devil to give decrepid old women power over the elements to produce lightnings and tempests, murrains and plagues; the woods and meadows were peopled with fairies, elves and goblins; the bones of long defunct saints wrought miracles and the Royal touch cured scrofula, (hence called King's evil) To the child who saw a witch hanged or burned for riding through the air on a broomstick, the sword of all-sharpness and the invisible cloak of Jack the Giant Killer presented no difficulties too great for acceptance, and the step from Jack the Giant Killer to David and Goliath was short and easy. Faith in the supernatural was universal and consistent, and the child grew into the man without being disillusionized. The beneficent miracles of the Bible were readily accepted by those who believed the malicious one of witchcraft. Faith, unquestioning faith, was the rule, and doubt and inquiry the exception, while church and state combined to convince the exceptional doubter, [with the gentle logic of the rack and stake,] of the wisdom and propriety of conforming to the rule.

The rising sun of knowledge has dissipated, in a great measure, the mists of superstition; we *know* now, that old women do not sail through the air on broomsticks, that Merlin and his magic palace, Aladdin and his wonderful lamp, Jack the Giant-killer, Cinderella and her Fairy Godmother are but the pithy creations of the poet's brain. But the charm of the world's old fables remains—childhood, still innocent and unsuspecting, listens and believes. In the nursery, the fairy wand and the magic slipper, Aladdin's lamp and the Genii of the lake are still rarities, and St. Nicholas with his annual visits down the chimney, as real as St. Peter or St. Paul. Then comes the awakening—the tender faith of childhood is shocked to learn that Santa Claus is a myth, and Jack the Giant-killer a fraud, that Cinderella's God-mother never did and never could transform, mice into horses, rats into coachmen, or pumpkins into golden chariots; unconsciously, the child reasons "if these things which I so fully believe are false, what *is*, what *can* be true. Shall I discard Jack and accept David, reject Cinderella's Fairy Godmother and accept the witch of Endor, pooh, pooh, at the wonders of the Arabian Nights and swallow the miracles of the Bible?" The old rule of evidence "false in one, false in all," is applied to the supernatural – the spirit of doubt, of inquiry, of skepticism in fact, is aroused never to be fully conquered.

If we wish our children to have faith, we must not begin by telling them lies, and after they have accepted them, admitting their falsity. If we wish them to *believe*, we must tell them as truth only what we believe to *be* truth.

Mankind, especially in childhood, is naturally credulous, naturally prone to believe in the marvellous and it is only after experience has taught us the falsity of many of our early beliefs that we become cautious about recieving statements upon mere authority, and instead of accepting everything with unquestioning faith, begin to doubt everything with undiscriminating skepticism—to believe nothing which can not be proved.

The cause, then, of skepticism is falsehood and deceit. The cure therefore, so far as cure is possible, must be truth and honesty. Let us be unwaveringly truthful with our children in regard to even the *little* things of this world, if we wish them to have faith concerning the great things of the next.

---

VACCINATION.—The question of the utility of vaccination which has been raised by the Anti-vaccination League in England, used by Mr. Bergh, and a few other erratic spirits in this country, must seem to any person of fair intelligence, who will take the trouble to investigate the matter fairly, as absurd as the assertion of the Rev. Brudder Jasper, of Richmond, Va., that "De sun *do move.*" Of course it is perfectly easy for the class of people who prepare their verdicts before hearing the evidence, to assert that vaccination is a useless protection against small pox, and is a fruitful source of injury to humanity by the introduction of the seeds of various other diseases along with the true vaccinia. The constant reiteration of such statements tends unfortunately to produce a distrust of vaccination in the minds of the general public who have neither time nor taste to investigate the matter for themselves.

In view of this fact it becomes pertinent at the present time to inquire:

1st. Does vaccination afford any protection against small pox ?

2nd. Is the protection, if any, permanent or temporary ?

3rd. Is there any serious danger to be apprehended from vaccination?

4th. Is the danger, if any, sufficient to counter-balance the protection afforded, if any?

The reply to the first question must be that *vaccination when properly performed, does afford protection against small-pox*, not only by preventing the disease but where it fails of this, modifying it, and mitigating its severer symptoms to so great an extent that it gets a new name—varioloid; and, persons suffering from it, though they may prove to be centers of infection from which true and deadly small-pox may be distributed, are themselves rarely seriously ill, and seldom carry through life any of the disfigurements in the way of pits and scars that so surely mark a person who has had small-pox unmodified by vaccination, when he been lucky enough to escape alive.

As to the question of permanence, the statistics of the Registrar General's office of Great Britain and the still more complete and accurate medical and sanitary records of the Prussian army demonstrate that in a certain number of cases the protection is permanent, while in other cases it appears to diminish with time, rendering successful revaccination possible and therefore necessary.

There is, as a rule, no serious danger to be apprehended from vaccination, though there are cases on record in which serious results have followed from simple wounds of no greater extent than the slight scratch, abrasion or puncture required by vaccination, and of course the presence of the vaccine virus in the wound can not afford protection against such accidents.

The introduction of other diseases can be perfectly provided against. 1st. By using only fresh lymph from a perfect vesicle on a healthy child.

2d. By using a perfect *recent* scab, free from blood.

3rd. By using lymph or scabs of non-humanized virus, carefully propagated on healthy calves—under the supervision of scientific and practical physicians.

This practically answers the fourth and last question, for, the danger of secondary complication of so slight a wound as is necessary for proper vaccination, are so slight as to be entirely without weight when compared to the protection afforded against one of the most loathsome and dangerous of diseases, which before the time of vaccination, was almost as fatal as the cholera or the plague, but since then has been reduced to a comparatively insignificant place among the "causes of death." Evidently just in proportion to the thoroughness with which proper vaccination has been enforced, communities in which vaccination has been nearly universal, have been practically free from small-pox, while other communities have suffered just in proportion to their neglect of vaccination.

Recent epidemics of small-pox have found their victims among the unvaccinated, or the imperfectly vaccinated.

Vaccination, then, *does* afford protection against small-pox—but it must be vaccination with proper matter and skillfully performed. Vaccine scabs which have become old, which contain blood, or which differ in anyway from the pure and perfect type, are useless and even dangerous, lymph or scabs from revaccination are useless—vaccination with perfect virus may be so unskillfully performed as to be of no avail, and this is doubtless the case with work of many amateur vaccinators, who of late, under the influence of fear or conceit, have undertaken the performance of this seemingly simple operation, of the details of which they know nothing, and have lulled their victims into false sense of security, affording opportunity for the anti-vaccination fanatics of the future to point out instances of the uselessness of vaccination.

The moral of all this is that, it is your duty to yourself, your neighbors and the State—to be vaccinated yourself, and have your children vaccinated by some reputable physician, and to have the operation repeated in from five to ten years. If it does not "take" the second time—well and good, if it does, its so doing proves that the vaccination was necessary.

